# Supplementary material for: The Effect of Anticoagulants, Temperature, and Time on the Human Plasma Metabolome and Lipidome from Healthy Donors as Determined by Liquid Chromatography-Mass Spectrometry
Source: Biomolecules. 2019 May 23;9(5):200. doi: 10.3390/biom9050200 (PMC6571950; doi:10.3390/biom9050200)

# Supplementary material 6: Base peak ion chromatogram for NIST SRM1950 in positive ionization mode metabolomics data

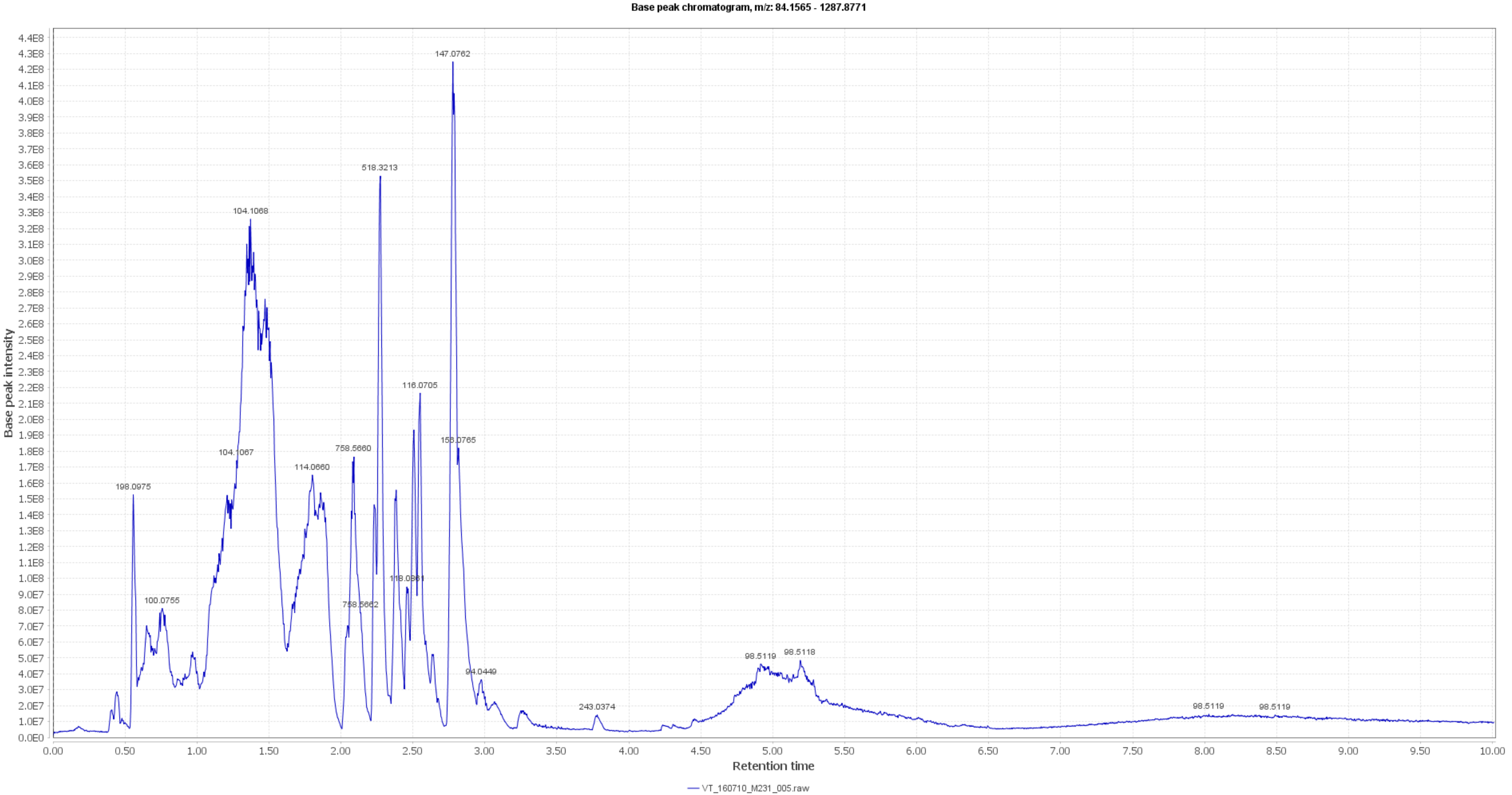

# Supplementary material 6: Base peak ion chromatogram for NIST SRM1950 in negative ionization mode metabolomics data

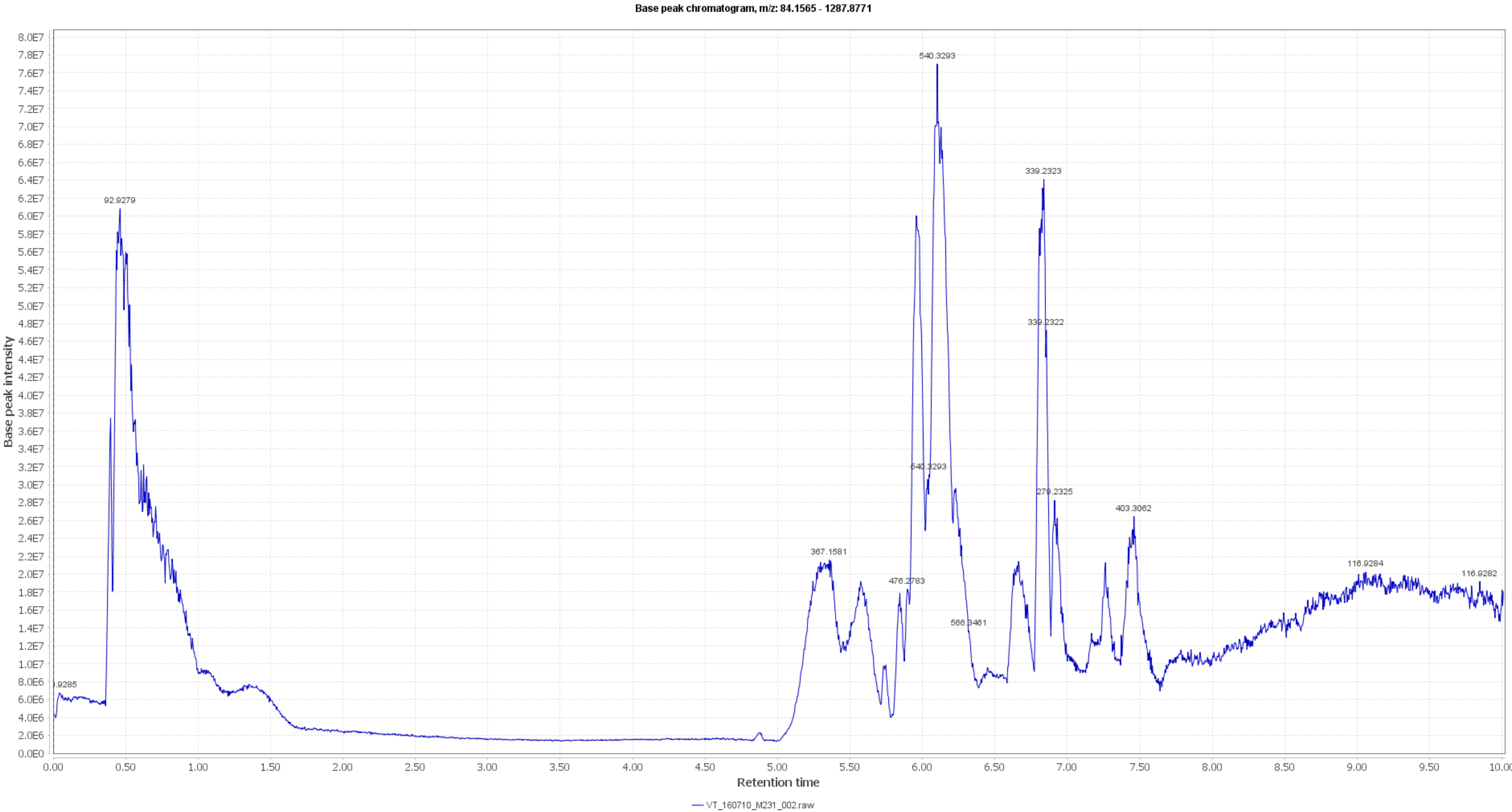

# Supplementary material 6: Base peak ion chromatogram for pooled plasma in positive ionization mode metabolomics data

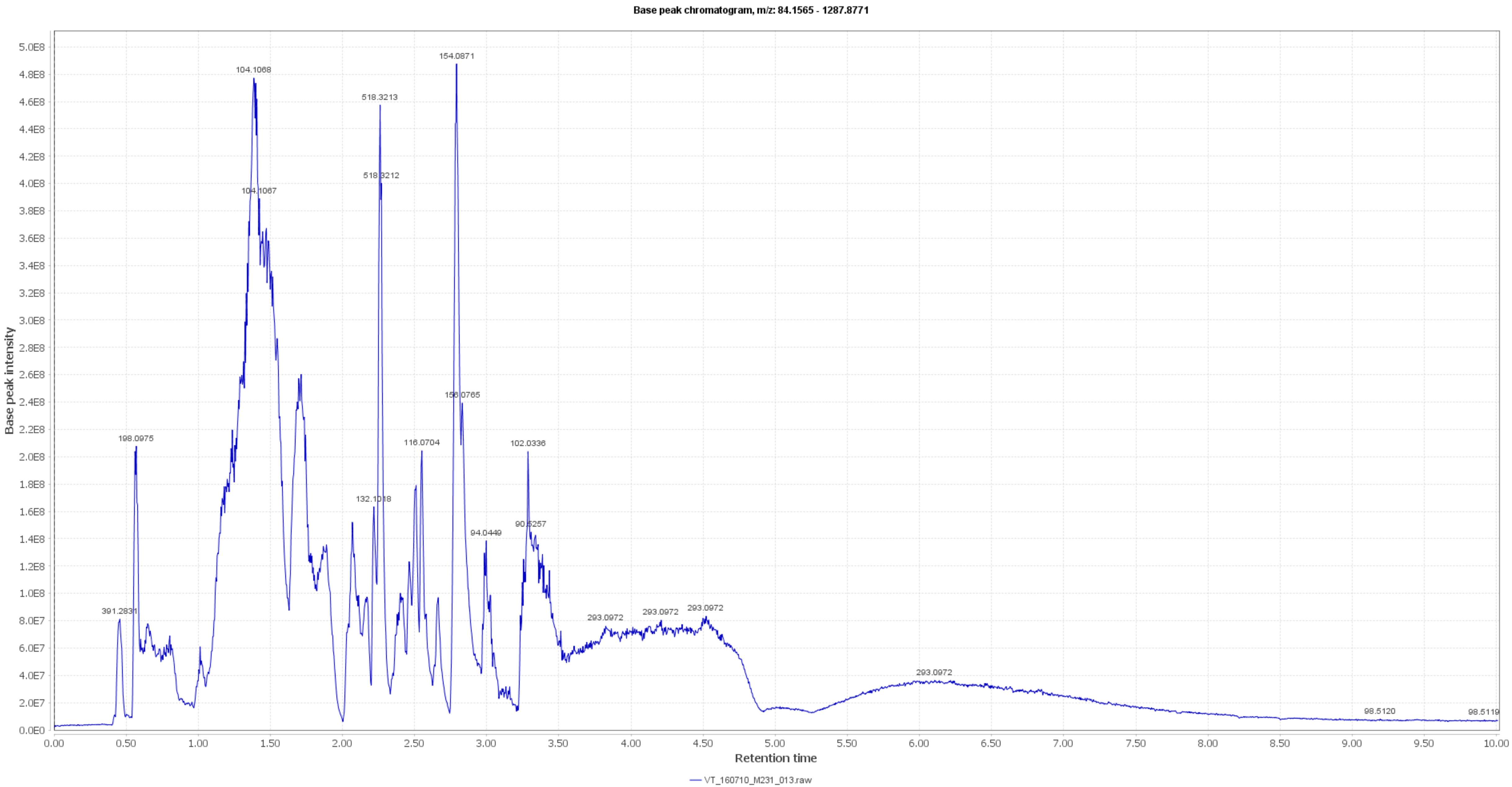

# Supplementary material 6: Base peak ion chromatogram for pooled plasma in negative ionization mode metabolomics data

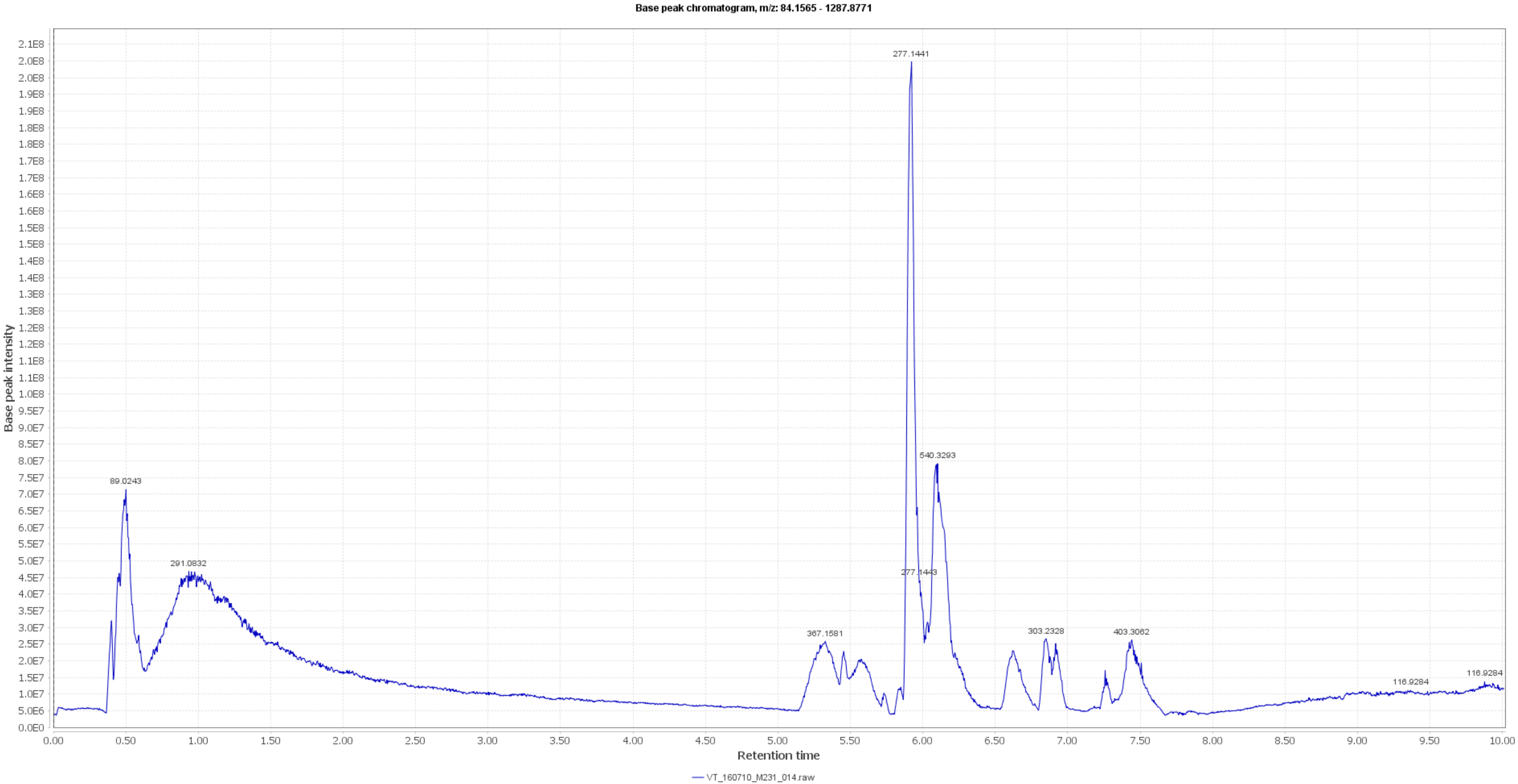

Supplement: Supplementary file 1 [file biomolecules-09-00200-s001.zip › Supplementary_material_6.pdf]
