# Supplementary material for: The Effect of Anticoagulants, Temperature, and Time on the Human Plasma Metabolome and Lipidome from Healthy Donors as Determined by Liquid Chromatography-Mass Spectrometry
Source: Biomolecules. 2019 May 23;9(5):200. doi: 10.3390/biom9050200 (PMC6571950; doi:10.3390/biom9050200)

Supplementary material 5: A) Base peak ion chromatogram representing separation of different lipid species in positive ionization mode

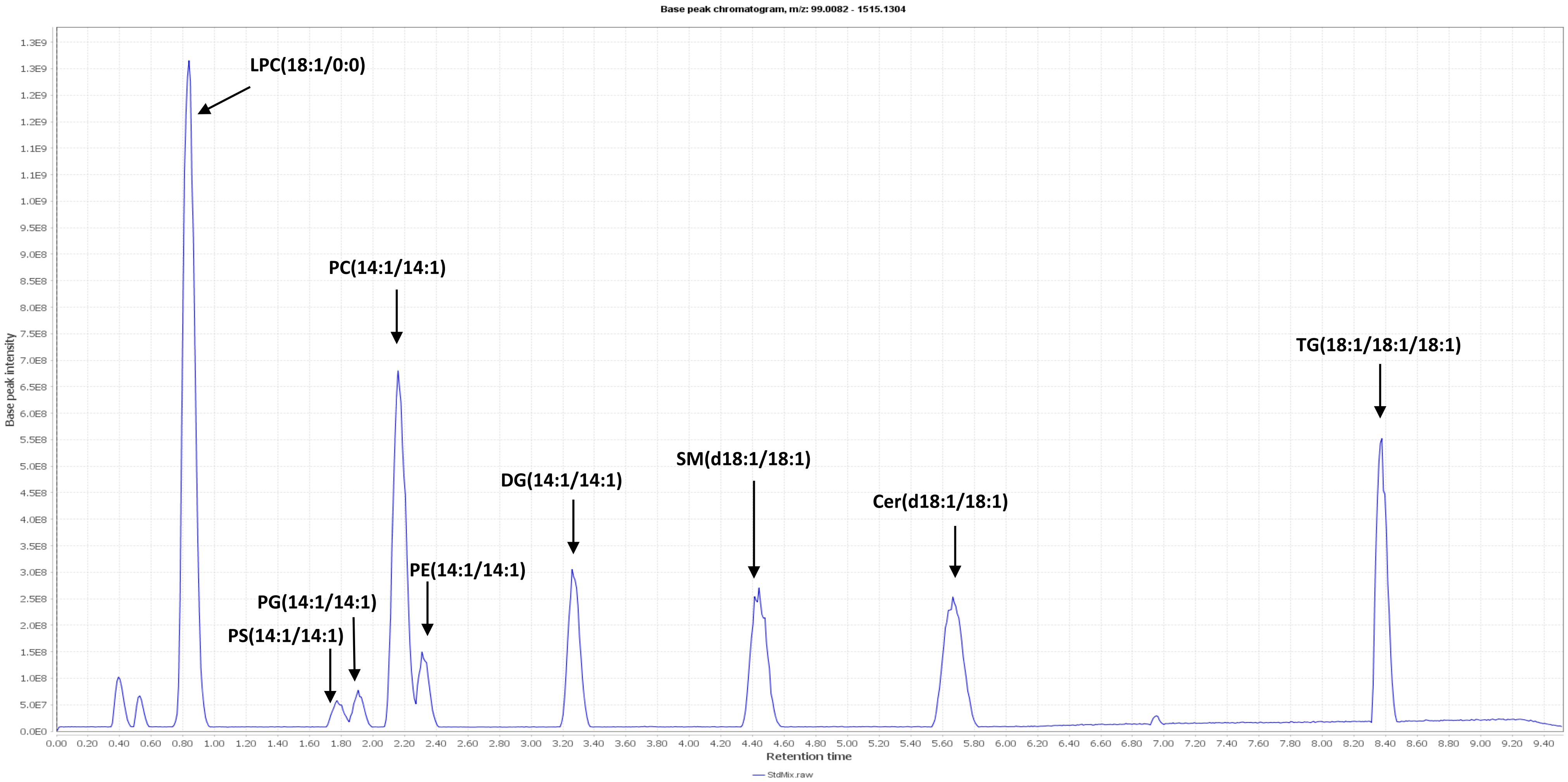

Supplementary material 5: B) Base peak ion chromatogram for D11_0H_CPT plasma in positive ionization mode lipidomics data

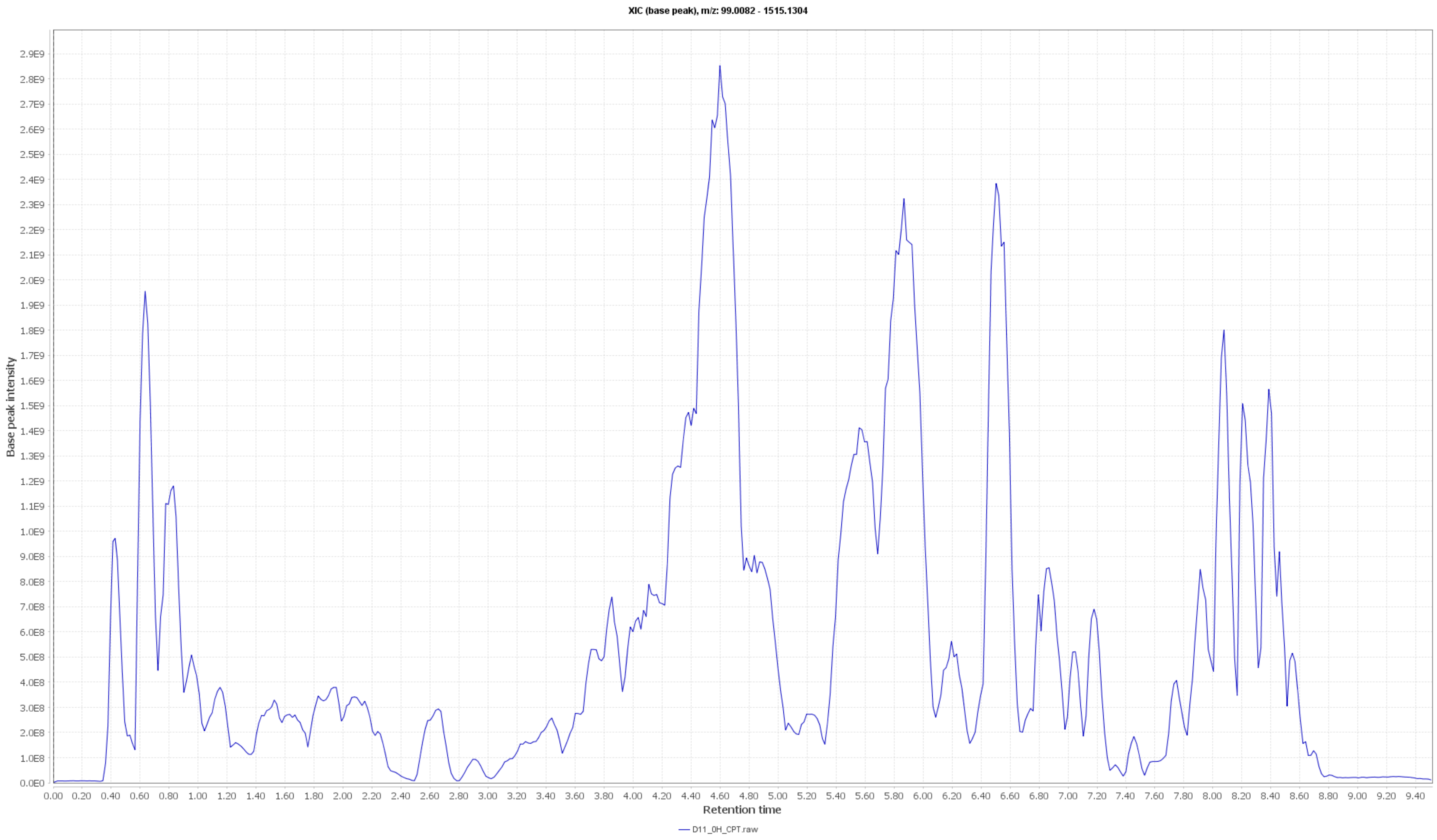

Supplementary material 5: C) Base peak ion chromatogram for D11_0H_CPT plasma in negative ionization mode lipidomicd data

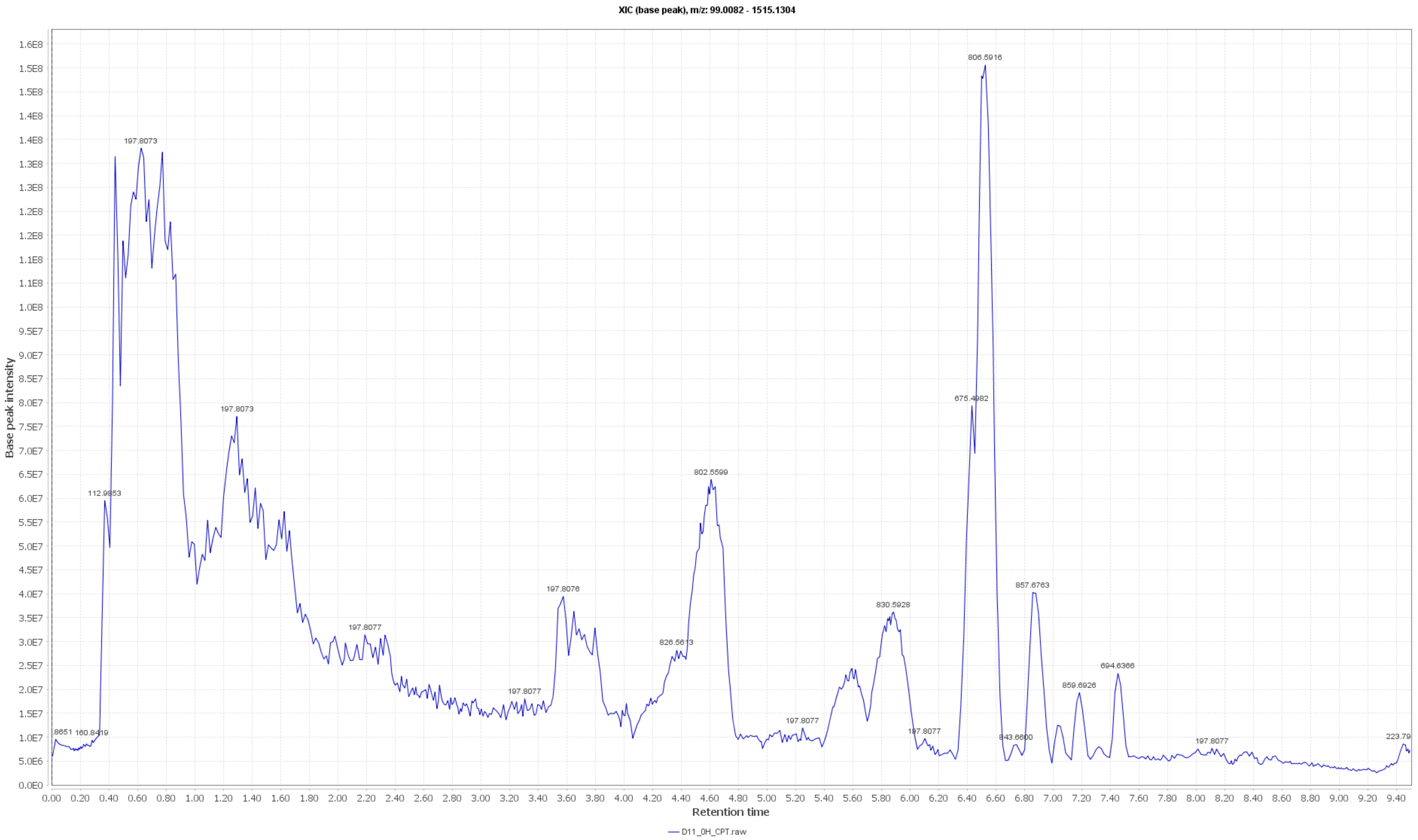

Supplement: Supplementary file 1 [file biomolecules-09-00200-s001.zip › Supplementary_material_5.pdf]
