# Supplementary material for: The Effect of Anticoagulants, Temperature, and Time on the Human Plasma Metabolome and Lipidome from Healthy Donors as Determined by Liquid Chromatography-Mass Spectrometry
Source: Biomolecules. 2019 May 23;9(5):200. doi: 10.3390/biom9050200 (PMC6571950; doi:10.3390/biom9050200)
Supplement: Supplementary file 1 [file biomolecules-09-00200-s001.zip › Supplementary_material_1.zip!/Supplementary_material_1/Readme.docx]

The supplementary files contains results showing the effect of anticoagulants in donor unmatched samples for positive mode lipidomics and metabolomics data.

File 1: One way ANOVA analysis with Fisher LSD test showing the effect of anticoagulants on positive mode lipidomics data in donor unmatched samples.

File 2: One way ANOVA analysis with Fisher LSD test showing the effect of anticoagulants on positive mode metabolomics data in donor unmatched samples.
