# Supplementary material for: The Effect of Anticoagulants, Temperature, and Time on the Human Plasma Metabolome and Lipidome from Healthy Donors as Determined by Liquid Chromatography-Mass Spectrometry
Source: Biomolecules. 2019 May 23;9(5):200. doi: 10.3390/biom9050200 (PMC6571950; doi:10.3390/biom9050200)
Supplement: Supplementary file 1 [file biomolecules-09-00200-s001.zip › Supplementary_material_2.zip!/Supplementary_material_2/Readme.docx]

The supplementary files are ANOVA analysis results to the study of the effect of anticoagulants in donor matched and unmatched samples in negative mode data from lipidomics and metabolomics studies. All the files are in .xlsx format.

SI 1: One-way ANOVA (FDR < 0.05) study showing all the significant features in donor unmatched samples grouped by anticoagulants in lipidomics study.

SI 2: One-way ANOVA (FDR < 0.05) study showing all the significant features in D11 samples grouped by anticoagulants in lipidomics study

SI 3: One-way ANOVA (FDR < 0.05) study showing all the significant features in D12 samples grouped by anticoagulants in lipidomics study

SI 4: One-way ANOVA (FDR < 0.05) study showing all the significant features in D13 samples grouped by anticoagulants in lipidomics study

SI 5: One-way ANOVA (FDR < 0.05) study showing all the significant features in D15 samples grouped by anticoagulants in lipidomics study

SI 6: One-way ANOVA (FDR < 0.05) study showing all the significant features in donor unmatched samples grouped by anticoagulants in metabolomics study.

SI 7: One-way ANOVA (FDR < 0.05) study showing all the significant features in D11 samples grouped by anticoagulants in metabolomics study

SI 8: One-way ANOVA (FDR < 0.05) study showing all the significant features in D12 samples grouped by anticoagulants in metabolomics study

SI 9: One-way ANOVA (FDR < 0.05) study showing all the significant features in D13 samples grouped by anticoagulants in metabolomics study

SI 10: One-way ANOVA (FDR < 0.05) study showing all the significant features in D14 samples grouped by anticoagulants in metabolomics study

SI 11: One-way ANOVA (FDR < 0.05) study showing all the significant features in D15 samples grouped by anticoagulants in metabolomics study
