# Supplementary material for: The Effect of Anticoagulants, Temperature, and Time on the Human Plasma Metabolome and Lipidome from Healthy Donors as Determined by Liquid Chromatography-Mass Spectrometry
Source: Biomolecules. 2019 May 23;9(5):200. doi: 10.3390/biom9050200 (PMC6571950; doi:10.3390/biom9050200)
Supplement: Supplementary file 1 [file biomolecules-09-00200-s001.zip › Supplementary_material_3.zip!/Supplementary_material_3/Readme.docx]

The supplementary files contain information on One way ANOVA analysis with Fisher LSD test results for the donor matched lipidomics and metabolomics positive mode data grouped by anticoagulants.

File 1: One way ANOVA with Fisher LSD for D11 lipidomics data grouped by anticoagulants

File 2: One way ANOVA with Fisher LSD for D12 lipidomics data grouped by anticoagulants

File 3: One way ANOVA with Fisher LSD for D13 lipidomics data grouped by anticoagulants

File 4: One way ANOVA with Fisher LSD for D15 lipidomics data grouped by anticoagulants

File 5: One way ANOVA with Fisher LSD for D11 metabolomics data grouped by anticoagulants

File 6: One way ANOVA with Fisher LSD for D12 metabolomics data grouped by anticoagulants

File 7: One way ANOVA with Fisher LSD for D13 metabolomics data grouped by anticoagulants

File 8: One way ANOVA with Fisher LSD for D14 metabolomics data grouped by anticoagulants

File 9: One way ANOVA with Fisher LSD for D15 metabolomics data grouped by anticoagulants
