# Supplementary material for: The Effect of Anticoagulants, Temperature, and Time on the Human Plasma Metabolome and Lipidome from Healthy Donors as Determined by Liquid Chromatography-Mass Spectrometry
Source: Biomolecules. 2019 May 23;9(5):200. doi: 10.3390/biom9050200 (PMC6571950; doi:10.3390/biom9050200)
Supplement: Supplementary file 1 [file biomolecules-09-00200-s001.zip › Supplementary_material_4.zip!/Supplementary_material_4/Readme.docx]

The supplementary files include One Way ANOVA analysis with Fisher LSD test for the donor matched lipidomics and metabolomics negative mode data grouped by storage time.

File 1: One way ANOVA with Fisher LSD test for the D11 negative mode lipidomics data grouped by storage time

File 2: One way ANOVA with Fisher LSD test for the D12 negative mode lipidomics data grouped by storage time

File 3: One way ANOVA with Fisher LSD test for the D15 negative mode lipidomics data grouped by storage time

File 4: One way ANOVA with Fisher LSD test for the D11 negative mode metabolomics data grouped by storage time

File 5: One way ANOVA with Fisher LSD test for the D12 negative mode metabolomics data grouped by storage time

File 6: One way ANOVA with Fisher LSD test for the D13 negative mode metabolomics data grouped by storage time

File 7: One way ANOVA with Fisher LSD test for the D14 negative mode metabolomics data grouped by storage time

File 8: One way ANOVA with Fisher LSD test for the D15 negative mode metabolomics data grouped by storage time
